# Quantitative Methods for Evaluating Antibody Responses to Pneumococcal Vaccines: A Scoping Review

**DOI:** 10.3390/tropicalmed10080236

**Published:** 2025-08-21

**Authors:** Yumiko Hayashi, Fleurette Mbuyakala Domai, Bhim Gopal Dhoubhadel

**Affiliations:** 1 School of Tropical Medicine and Global Health, Nagasaki University, Nagasaki 852-8523, Japan; 2 Department of Respiratory Infections, Institute of Tropical Medicine, Nagasaki University, Nagasaki 852-8523, Japan; bhimgopal@gmail.com; 3 Graduate School of Biomedical Sciences, Nagasaki University, Nagasaki 852-8523, Japan; fleurbydo@gmail.com

**Keywords:** *Streptococcus pneumoniae*, vaccines, antibody assessments, immunoassays, vaccine protection

## Abstract

*Streptococcus pneumoniae* remains a leading cause of invasive diseases, particularly affecting young children and the elderly. Currently, two main types of pneumococcal vaccines are commercially available: polysaccharide vaccine (PPSV23) and conjugate vaccines (e.g., PCV20). Of over 100 identified pneumococcal serotypes, vaccines targeting 24 serotypes covered by PPSV23 and PCV20 (19 serotypes overlap between the two vaccines) have been developed, with serotype distribution varying by geography, age, and time. The immune response to pneumococcal vaccines differs across serotypes, vaccine types (polysaccharide vs. conjugate), and host factors. Quantitative methods for antibody assessment—particularly newer high-throughput assays—have emerged since 2000 to address limitations in conventional approaches. However, these methods have not been comprehensively reviewed. This scoping review aimed to systematically map the existing literature on quantitative methods used to assess antibody responses to pneumococcal vaccines. Specific objectives included the following: 1. summarizing conventional and novel quantitative immunoassays; 2. evaluating the current state of validation and application of these methods; 3. identifying knowledge gaps and methodological challenges. We followed the PRISMA-ScR guidelines. We included the following: 1. peer-reviewed, open-access papers related to immunoassays used for pneumococcal antibody assessment; 2. articles written in English; 3. Studies published between 2000 and 2023. We excluded the following: 4. studies focusing on other pathogens, employing different analytical methods, or using animal models. Articles meeting the eligibility criteria were primarily retrieved from PubMed and Scopus. If free full-text versions were unavailable there, Google Scholar or the original journal databases were consulted. All references were exported to EndNote 20 for further management. At the beginning of the review, a data-charting form was developed based on prior studies and commonly addressed themes. Additional charts were created to accommodate newly identified variables during the review. All charting tools were reviewed and finalized through discussion among all research team members. The included studies were classified into five thematic groups: 1. general descriptions of quantitative assessment methods, 2. assay development and validation, 3. comparative studies, 4. technical details of assay development, 5. interpretation of assay application findings. Of 1469 articles from PubMed and 2946 articles from Scopus initially identified, 55 articles met the inclusion criteria. The earliest methods included radioimmunoassays, later replaced by WHO-standardized ELISA. While ELISA remains the gold standard, it is limited by labor, cost, and throughput. Multiplex immunoassays (MIAs), including Luminex-based platforms, have demonstrated advantages in efficiency and scalability. However, many MIAs did not initially meet WHO validation criteria. More recent assays show an improved performance, yet interlaboratory variability and lack of standardized protective thresholds remain major limitations. This review provides the first comprehensive mapping of quantitative antibody assessment methods for pneumococcal vaccines. Although ELISA continues to serve as the benchmark, MIAs represent a promising next-generation approach. Continued efforts are needed to harmonize assay validation protocols and establish global standards for protective thresholds, which will enhance the reliability of vaccine efficacy monitoring across diverse populations.

## 1. Introduction

*Streptococcus pneumoniae* (pneumococcus) is a major causative agent of pneumonia and invasive diseases, particularly affecting young children and older adults due to their immature or weakened immune systems [1,2,3]. The pathogen’s polysaccharide capsule varies structurally, resulting in over 100 identified serotypes [4]. These serotype distributions differ by geography, age, and time [5,6], with specific serotypes such as 1, 5, 7F, 8, 14, 18C, 33F, and 38 commonly associated with invasive pneumococcal diseases [3]. This variability informs vaccine formulation strategies to target the most relevant serotypes in different populations.

Pneumococcal vaccines are classified into two main types: polysaccharide vaccines and conjugate vaccines. The 23-valent pneumococcal polysaccharide vaccine (PPSV23) has been licensed since 1983 [7,8], while the 7-valent conjugate vaccine (PCV7) was introduced for children under five in 2000 [9]. Subsequent formulations—PCV10 and PCV13—were introduced in 2007 and 2010, respectively [6]. More recently, PCV15 and PCV20 have become available in high-income countries including the U.S., Europe, and Japan [10,11,12,13,14].

Initial quantification of pneumococcal antibody responses relied on the radioimmunoassay (RIA), which was widely used in the 1980s [15]. Due to the use of radioactive materials, RIA was replaced by enzyme-linked immunosorbent assay (ELISA), a safer and more accessible method [16,17]. Currently, the WHO recommends assessing IgG concentrations using ELISA as a primary analysis and evaluating functional antibody activity via opsonophagocytic assays [18]. The WHO-established threshold of 0.35 μg/mL IgG is considered protective against invasive pneumococcal disease (IPD) in children vaccinated with conjugate vaccines [18,19,20]. However, recent studies suggest that protective levels vary by serotype, underscoring the need for serotype-specific assessments [21].

**Figure 1:**
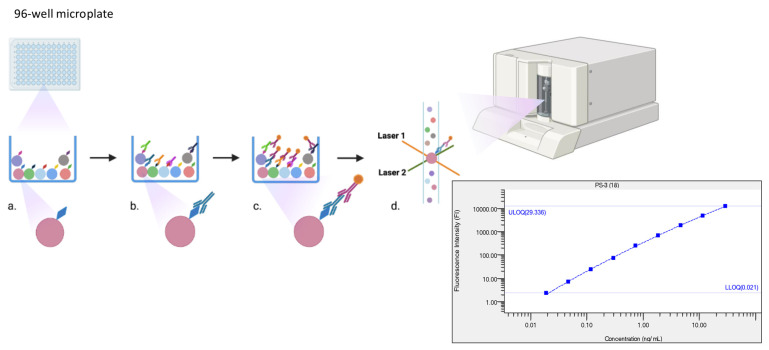
Principle of a multiplex immunoassay using BioRad 200 system. (**a**) Conjugation of microsphere beads with antigens; (**b**) primary antibody and antigen binding; (**c**) secondary antibody with a reporter binding with primary antibody; (**d**) detection of color of microsphere beads and fluoresces of reporter by lasers 1 and 2 in the BioRad 200 system and quantification of antibody concentration using a standard curve.

Despite its robustness, ELISA has limitations in terms of throughput, labor, time, and cost. In contrast, multiplex immunoassays (MIAs), including Luminex-based systems, offer high-throughput analysis capable of quantifying antibodies against up to 100 serotypes in a single run (Bio-Rad, Hercules, CA, USA) [22] (Figure 1). Several research groups have developed MIAs that show promising agreement with WHO standards [23,24,25]. Nonetheless, comprehensive reviews of their development, validation, and application remain lacking.

### Objectives

This scoping review aims to systematically map the existing literature on quantitative methods used to assess antibody responses to pneumococcal vaccine antigens. Specifically, the objectives are as follows:

1. To identify and categorize conventional and emerging immunoassays, including ELISA and multiplex immunoassays (MIAs), applied in the quantitative evaluation of pneumococcal antibodies;
2. To evaluate the extent of assay development, validation, and standardization reported across studies;
3. To highlight methodological challenges and gaps in the current literature that may inform future improvements in pneumococcal vaccine immunogenicity assessment.

## 2. Methods

### 2.1. Protocol and Registration

This scoping review was developed in accordance with the Preferred Reporting Items for Systematic Reviews and Meta-Analyses Protocols (PRISMA-P). The research protocol was drafted and revised by the research team at the Department of Respiratory Infections, Institute of Tropical Medicine, Nagasaki University, Japan. The final version was registered with the Open Science Framework on 18 January 2024 (Registration DOI: https://doi.org/10.17605/OSF.IO/DY3FM).

### 2.2. Eligibility Criteria

We included literature that met the following criteria:

- Publication type: Peer-reviewed original articles, reviews, editorials, letters, and reports.
- Language: English.
- Timeframe: Published between 2000 and 2023.
- Content focus: Quantitative assessment of pneumococcal vaccine antibody responses via immunoassays, including assay development, validation, and application in human samples.

Exclusion criteria included the following:

- Articles not in English;
- Publications outside the designated time frame;
- Studies focusing on non-pneumococcal pathogens, non-capsular antigens, non-quantitative methods, or animal models;
- Articles without peer review or lacking free access.

A summary of inclusion and exclusion criteria is presented in Table 1.

### 2.3. Information Sources

We conducted an initial search in PubMed and Scopus to identify relevant studies. If full-text access was not available, additional sources such as Google Scholar and original databases (e.g., WHO Technical Report Series) were used. Selected references were exported to EndNote 20 for screening and citation management. We used Microsoft^®^ Excel for Mac (version 16.99.2) for mapping the extracted information and the PRISMA-ScR check list provided by the PRISMA official site (https://www.equator-network.org/wp-content/uploads/2018/09/PRISMA-ScR-Fillable-Checklist.pdf, accessed on 8 July 2025).

### 2.4. Search Strategy

The search strategy included combinations of the following keywords:

1. Organism: “*Streptococcus pneumoniae*” or “pneumococc*”;
2. Methodology: “multiplex”, “Luminex”, “ELISA”, or “enzyme-linked immunosorbent assay”;
3. Immunological terms: “pneumococcal IgG” or “pneumococcal antibody”.

Search terms were grouped using Boolean operators and applied to titles and abstracts in PubMed and Scopus. The number of articles retrieved per term was assessed, and a pre-screening selection of studies containing all relevant terms was conducted (Supplementary File S1).

### 2.5. Selection of Sources of Evidence

Articles were initially screened by title and abstract. Full-text screening was conducted for those that passed the initial phase. Studies that failed to meet the eligibility criteria were excluded during full-text review.

### 2.6. Data-Charting Process

A standardized data-charting form was developed based on the existing literature and refined collaboratively by the research team: two reviewers in the team. Additional charts were created for further sub-group analyses. All the selected articles were assessed and the charts were discussed until reaching consensus by the members. If the two members did not reach consensus, the other member assessed the controversial articles according to the eligibility criteria.

### 2.7. Data Items

For each included study, the following data were extracted:

- Author(s), year, title, journal/source, DOI;
- Type of study/article;
- Assay type, number of serotypes assessed, sample type and size;
- Validation metrics (e.g., specificity, sensitivity, reproducibility);
- Key findings related to assay development, evaluation, and application.

### 2.8. Synthesis of Results

Studies were categorized into five thematic groups based on content:

1. General descriptions of quantitative assessment methods (Group 1);
2. Assay development and validation (Group 2);
3. Comparative studies of different assays (Group 3);
4. Technical descriptions of assay development (Group 4);
5. Interpretation and application of findings (Group 5).

Studies that addressed multiple themes were classified under more than one category. Group 1 was used to provide historical and conceptual context, while Groups 4 and 5 were used to identify methodological challenges and research gaps.

## 3. Results

### 3.1. Sources of Evidence

**Figure 2:**
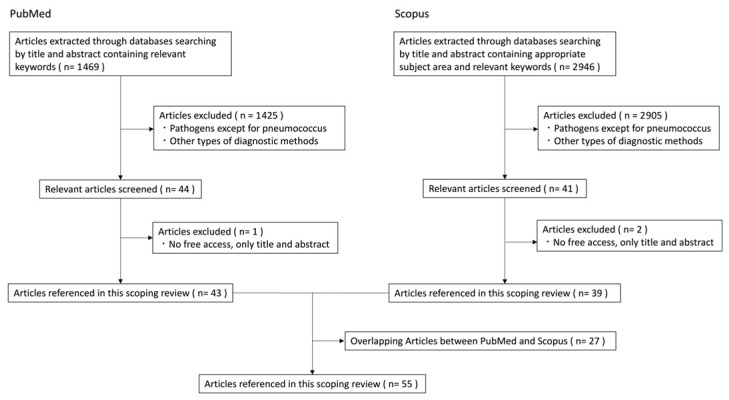
Flow of selection of articles.

A total of 1469 articles from PubMed and 2946 articles from Scopus were identified through these databases using the defined search strategy. After excluding 1425 articles and 2905 articles, respectively, that focused on non-pneumococcal pathogens, irrelevant diagnostic methods (e.g., multiplex PCR for DNA detection, antigen detection in urine), or animal studies, 44 articles from PubMed and 41 articles from Scopus were selected for full-text review. Three articles were excluded due to lack of full-text access. Furthermore, 27 articles overlapped between the two database. Ultimately, 55 articles were included as sources of evidence in this scoping review (Figure 2; Supplementary File S2).

### 3.2. Characteristics of Sources of Evidence

The selected studies varied in focus and type, with some being narrative in nature (e.g., reviews, reports, letters) and others based on laboratory research or clinical studies. Studies categorized under “general descriptions” (Group 1), “technical details” (Group 4), and “interpretation of findings” (Group 5) tended to be more descriptive, while those under “assay development and validation” (Group 2) and “comparative studies” (Group 3) were largely empirical.

### 3.3. Results of Individual Sources of Evidence

The 55 studies were categorized into the following groups:

- Group 1: General descriptions of quantitative assessment methods (n = 8);
- Group 2: Assay development and validation (n = 11);
- Group 3: Comparative studies (n = 18);
- Group 4: Technical development of assays (n = 10);
- Group 5: Interpretation and application of findings (n = 25).

Note: Some studies were allocated to more than one group if they addressed multiple themes.

#### Group 1: General Description of Quantitative Assessment Methods

Several studies described the historical evolution of quantitative assays, from early radioimmunoassay (RIA) in the 1980s [15] to the adoption of enzyme-linked immunosorbent assay (ELISA) as a safer alternative [16,17,26]. Inter-laboratory evaluations were conducted in Europe and the U.S. to improve reproducibility [27]. By the early 2000s, a third-generation ELISA had emerged as the WHO gold standard, following improvements in assay specificity [16]. Since then, several research groups have focused on developing multiplex immunoassays (MIAs) as a high-throughput alternative [28,29].

### 3.4. Principles of ELISA and MIA

ELISA relies on coating 96-well plates with one type of serotype-specific antigen. Upon the addition of serum samples, antibodies bind to the antigen, and the reaction is measured using a fluorescence signal [18,29]. MIA, by contrast, employs microspheres conjugated with multiple serotype-specific antigens. Each microsphere is uniquely color-coded, allowing simultaneous detection of antibodies against up to 100 serotypes within a single well [22]. This makes MIA significantly more efficient than traditional ELISA.

#### 3.4.1. Group 2: Assay Development and Validation

Eleven studies focused on the development and validation of quantitative immunoassays (Table 2). RIA was the earliest method applied for pneumococcal antibody measurement [15], but safety concerns led to the development of ELISA. Several studies evaluated ELISA’s accuracy and cross-reactivity, culminating in efforts to standardize assay protocols [27,28,30,31,32,33,34]. With the WHO’s 2013 recommendations for assay validation, an emphasis was placed on eight key performance metrics: specificity, accuracy, precision, detection limit, quantitation limit, linearity, range, and robustness [18]. MIAs developed in the early 2000s by Pickering et al. [28], Biagini et al. [23], Lal et al. [24], Klein et al. [35], and Whitelegg et al. [36] demonstrated increased throughput but often did not meet all WHO validation criteria. Notably, Marchese et al. [25] developed a multiplex electrochemiluminescence (ECL)-based assay that met WHO standards. Subsequent developments by Pavliakova et al. [37] and Nolan et al. [38] further improved validation, demonstrating acceptable specificity, linearity, and reproducibility across multiple serotypes using international reference sera.

#### 3.4.2. Group 3: Comparative Studies

Eighteen articles compared MIA with WHO reference ELISA methods (Table 3). Most studies were conducted in the U.S. and Europe, assessing between 7 and 23 serotypes with sample sizes ranging from 10 to 1528. Nine studies reported strong correlations or non-inferiority of MIA relative to ELISA, while four studies found discordant results, particularly for pre-immunization and pediatric samples. Plikaytis et al. [27] demonstrated strong inter-laboratory consistency for ELISA, supporting its adoption as a global reference method. More recently, European multi-center studies confirmed the reproducibility of MIA, although serotype-specific variability remains a challenge [39].

#### 3.4.3. Group 4: Technical Description of Assay Development

Ten studies addressed specific technical aspects of assay development (Supplementary File S2). Park et al. [49] conducted pioneering research on the development of bead-based flow cytometric immunoassay around 2000. To improve specificity, several studies optimized adsorption techniques using non-target serotype polysaccharides—such as types 22F, 25, and 72—to reduce cross-reactivity [28,33,50]. Coupling chemistry was another focus. Schlottmann et al. [51] compared oxidation, poly-L-lysine (PLL), and COOH-DMTMM methods, identifying the most effective for conjugating polysaccharides to microspheres. Hansenova et al. [52] highlighted the enhanced performance of magnetic over non-magnetic beads. Daly et al. [53] explored measurement approaches, concluding that fold-change algorithms reduced variability better than threshold-based methods.

#### 3.4.4. Group 5: Interpretation and Application of Assay Findings

Twenty five studies examined how validated assays were applied in practice (Supplementary File S2). Some evaluated antibody function using opsonophagocytic activity, while others explored setting protective cut-off values. Siber et al. [32] described how to estimate protective levels with ELISA, and more recent studies sought to translate this threshold into MIA platforms [47,54]. While MIAs offer advantages, studies noted variability in antibody levels across platforms and uncertainty about applying the WHO’s 0.35 μg/mL cut-off to MIA data [42,45,53,55]. These findings underscore the need for unified interpretation frameworks tailored to MIA results. However, MIA platforms have already been applied to the evaluation and comparison of antibody levels for PPSV23 and conventionally used conjugate vaccines [56,57,58,59,60,61]. Currently it has also applied for several clinical studies to assess newly developed vaccines such as PCV15 and PCV21 [62,63,64].

## 4. Discussion

### 4.1. Summary of Evidence

In this scoping review, we examined 55 studies published between 2000 and 2023 on quantitative methods for assessing antibody responses to pneumococcal vaccines. Our analysis highlights the evolution of immunoassay techniques from radioimmunoassay (RIA) to enzyme-linked immunosorbent assay (ELISA), and more recently, to multiplex immunoassays (MIAs). Since the early 2000s, MIAs have gained attention as next-generation tools for simultaneously evaluating antibody responses to multiple pneumococcal serotypes, offering notable advantages in cost, time, and sample efficiency. Despite these benefits, MIAs face challenges such as inter-laboratory variability and a lack of universally accepted protective thresholds. As a result, ELISA remains the gold standard for clinical and regulatory use.

### 4.2. ELISA vs. MIA: Comparative Perspectives

Many studies have acknowledged the limitations of ELISA, particularly its inefficiency in simultaneously assessing multiple serotypes. ELISA requires one assay per serotype, increasing time, sample volume, and cost proportionally [17,65,66]. In contrast, MIA enables parallel analysis of multiple serotypes within a single run, reducing running costs by up to 89%, sample volume by 80%, and experimental time by 92% [23,24,28,65].

### 4.3. Assay Validation and WHO Standards

Validation is essential before applying assays to clinical or research settings. In 2013, the WHO outlined eight recommended validation criteria: specificity, accuracy, precision, detection limit, quantitation limit, linearity, range, and robustness [18]. Prior to this, studies evaluated only a subset of these parameters [23,28,35,36]. Recent studies, however, have adhered more closely to WHO guidelines and reported successful validation across most parameters [25,37,38] (Table 4).

One critical distinction between ELISA and MIA lies in their dynamic range. ELISA typically provides a ~10-fold quantifiable range, while MIAs can achieve ranges from 24-fold to over 500-fold. ELISA results are usually reported in μg/mL, whereas MIAs often use more sensitive units such as ng/mL or pg/mL. Although many MIAs maintain a strong correlation with ELISA, inconsistencies in validation methodologies and reporting formats hinder direct comparability across studies.

### 4.4. Protective Thresholds and Remaining Challenges

The WHO reference ELISA established a protective IgG threshold of 0.35 μg/mL for assessing the risk of IPD in vaccinated children [27,32,50]. However, this threshold applies specifically to children vaccinated with PCVs and may not be universally appropriate. It also does not account for variability among serotypes, vaccine formulations, or population-specific immunological factors [18,19,20]. These limitations complicate the application of a unified standard to MIAs, particularly when platforms use differing conjugation chemistries and laboratory protocols. Consequently, inter-laboratory discrepancies and systematic bias remain major barriers to harmonization. Bridging MIA outputs to the WHO reference ELISA requires a careful alignment with established validation frameworks. A uniform validation protocol, combined with cumulative data from previous MIA studies, may facilitate the development of standardized, next-generation immunoassay protocols.

### 4.5. Limitations

Although this scoping review aimed to comprehensively map the available literature, some relevant studies may have been missed. Unlike a systematic review, a scoping review is characterized by the overarching screening approach. Therefore, a rigorous evaluation of individual studies was not conducted. Consequently, we should consider a thorough evaluation of the methodological validity within and across the included studies as a task for future work. We also noted potential duplication among reports from the same research groups, which was difficult to resolve in the absence of a centralized registry. Furthermore, comparing assay performance across studies was challenging due to inconsistent validation methods and reporting formats. Serotype-specific thresholds should be established, and the WHO has proposed validation criteria for bridging studies. However, the evaluation parameters vary across studies, and no assay system has yet been established to replace the WHO ELISA, which could define serotype-specific thresholds. Therefore, it is difficult to determine thresholds for each serotype at present. To address these challenges, an international registry for pneumococcal antibody assay protocols and validation data may help streamline assay comparisons and support the development of new global standards.

## 5. Conclusions

This scoping review provides the first comprehensive mapping of quantitative methods used to evaluate antibody responses to pneumococcal vaccines. While many of the existing studies are dispersed and descriptive, this review synthesizes them into a structured framework that clarifies both methodological advancements and current limitations. Although the WHO reference ELISA remains the established standard for quantitative antibody assessment, multiplex immunoassays (MIAs) are emerging as promising next-generation alternatives. MIAs offer significant advantages in throughput, efficiency, and resource utilization. Importantly, several MIAs have now met WHO validation criteria and demonstrated reproducibility across laboratories. Nonetheless, unresolved issues remain—particularly the lack of standardized protective thresholds and persistent inter-laboratory variability.

To advance the field, it is essential to establish an international, systematic database of validated MIA protocols and results. Such a repository would facilitate a comparison, support the harmonization of methodologies, and enable the future development of a WHO-standardized MIA protocol. Once standardized, MIAs could enable serotype-specific threshold setting, informed vaccine selection, and robust immunogenicity monitoring across diverse populations and settings.

## Figures and Tables

**Table 1:** Eligibility criteria summary.

Criterion	Inclusion	Exclusion
Publication type	Peer-reviewed original articles, reviews, editorials, letters, reports	Non-peer-reviewed reports, commentaries
Language	English	Other languages
Time frame	2000–2023	Published before 2000 or after 2023
Focus	Quantitative pneumococcal antibody immunoassays: development, validation, use	Animal studies, non-capsular antigens, PCR-based methods, non-quantitative work
Accessibility	Full text available	Full text unavailable

**Table 2:** Summary of assay development and validation studies.

Study	Study Type	Assay Type	No. of Serotypes	Sample Type	Validation Items	Key Findings
Black S, et al. [30] (2000)	Double-blind, randomized control trial	ELISA	7	Infant serum vaccinated with PCV7	Efficacy analysis by active surveillance for cases of invasive pneumococcal disease in the study population, immunogenicity by serum antibody responses (IgG) to the vaccine serotypes	More than one dose of 7-valent pneumococcal conjugate vaccine for children reduced 89.1% of the total IPD burden. All seven serotypes showed immunologic response to pneumococcal polysaccharide. This study predicted the minimum antibody titer of post-primary doses related to long-term protection against IPD; it was between 0.15 and 0.5 μg/mL with Wyeth ELISA.
Pickering JW, et al. [28] (2002)	Comparative study-Comparison multiplex immunoassay (MIA) with standard ELISA	MIA	14	For establishing the assay: U.S. reference standard serum (89SF). For application: 50 pneumococcal quality control serum samples	Specificity, concordance with ELISA	The authors investigated the specificity of 14 serotypes with multiplex immunoassay. Inhibition by homologous serotypes was more than 95% and inhibition by heterologous serotypes was less than 15% for all 14 PnPs serotypes.
Biagini RE, et al. [23] (2003)	Laboratory research	MIA	24	U.S. reference standard serum (89SF)	Linearity range (sensitivity), adsorption, specificity	Wider linearity than ELISA on dilution across the dynamic range between 20 pg/mL for PnPS 3 and 600 pg/mL for PnPS 14.
Lal G, et al. [24] (2005)	Comparative study-Comparison MIA with standard ELISA	MIA	9	For establishing the assay: U.S. reference standard serum (89SF). For application: a panel of sera (n = 120)	Specificity, sensitivity, accuracy, reagent stability	The multiplex assay generated a linear dynamic range over a 24-fold serum dilution and did not show bead interference compared to monoplex and nonaplex assays. The assay specificity was <30% with heterologous inhibition and the sensitivity was high, with the limit of detection ranging between 32.3 and 109.7 pg/mL. Inter- and intra-assay variability was low. Conjugated microspheres were stable over 12 months.
Marchese RD, et al. [33] (2006)	Comparative study-Comparison in-house ELISA with standard ELISA	ELISA	12	U.S. reference standard serum (89SF)	Limit of detection (LOD) and limit of quantitation (LOQ), specificity, precision	Serum preadsorption with capsular polysaccharides, serotype 25 and 72 pneumococcal polysaccharides, was implemented. It reduced non-specific cross-reaction. The ELISAs demonstrate agreement with the internationally accepted ELISA.
Marchese RD, et al. [25] (2009)	Comparative study-Comparison multiplex electrochemiluminescence (ECL) assay with standard ELISA	Multiplex ECL assay (MIA)	23	For establishing the assay: U.S. reference standard serum (89SF). For application: WHO quality control panel	Standard curve modeling, quantifiable range, ruggedness, precision, dilutability, specificity, accuracy, effect of preadsorbent	The multiplex ECL assay shows a wide dynamic range and provides the ability to read lower concentrations to the minimum reported concentration than ELISA (0.1 μg/mL). Cross-reactivity assessment showed no cross reaction between antigen spots within a well.
Klein DL, et al. [35] (2012)	Comparative study-Comparison MIA with standard ELISA	MIA	7	For establishing the assay: U.S. reference standard serum (89SF). For application: paired sera from pregnant women who received one dose of PPSV23	Limit of detection (LOD), specificity, robustness, correlation of two assays, reproducibility	The developed assay showed good robustness, with the assay variability at <16%. A major contributor to the assay variability was the cell wall polysaccharide content in pneumococcal serotype-specific capsular polysaccharides. This assay is specific, robust, and reproducible and offers high throughput.
Whitelegg AME, et al. [36] (2012)	Laboratory research	MIA	13	For establishing the assay: U.S. reference standard serum (89SF). For application: NHSBT sera (n = 193)	Specificity, reproducibility	Serotype-specific IgG mean fluorescence intensities remained above 60% except for serotype 7F after preabsorption with each heterologous antigens. Intra-assay coefficient of variation for specific IgG ranged between 4% and 25%. Inter-assay coefficient of variation was between 17% and 57%.
Lee H, et al. [34] (2017)	Laboratory research	ELISA	7	Serum reference standard (89SF)	Specificity, precision (reproducibility and intermediate precision), accuracy, spiking recovery test, lower limit of quantification (LLOQ), and stability	The specificity, reproducibility, and intermediate precision were within acceptance ranges (reproducibility, coefficient of variability [CV] ≤ 15%; intermediate precision, CV ≤ 20%) for all serotypes.
Pavliakova D, et al. [37] (2018)	Laboratory research	MIA	13	International reference serum (007sp)	Linearity range (sensitivity), specificity, precision, accuracy, and stability	The authors remarked on the importance of this study: (i) validating the assay and (ii) bridging the immunological correlation of the protective level (0.35 μg/mL IgG) to equivalent values reported by the Luminex platform. The lower limit of quantitation for all serotypes covered in this developed assay was within the range of 0.002 to 0.038 μg/mL serum IgG. The difference between the lower and upper limits of the range was >500-fold for all serotypes, and assay variability was <20% relative standard deviation for all serotypes.
Nolan KM, et al. [38] (2020)	Laboratory research	Multiplex ECL assay (MIA)	15	International reference serum (007sp)	Limit of detection (LOD), quantifiable range (limits of quantitation; LOQs), precision, accuracy, specificity, selectivity, and dilutional linearity	This study bridged the multiplex ECL assay with the WHO ELISA. All validation items were evaluated according to the pre-specified validation acceptance criteria, and all of them met the criteria.

**Table 3:** Comparative studies of assays for pneumococcal antibody assessment.

Study (Year)	Study Site	Study Type	Assay Type	No. of Serotypes	Sample Type	Sample Size	Key Findings
Plikaytis BD, et al. [27] (2000)	US, France, Denmark, UK, Iceland, Finland	Inter-lab comparison	ELISA	9	Paired adult serum pre- and post-vaccination of PPSV23 (quality control sera)	48	High inter-lab concordance (Spearman ≥ 0.92); validated WHO ELISA.
Pickering JW, et al. [28] (2002)	US	Comparative	MIA vs. ELISA	14	For establishing the assay: U.S. reference standard serum (89SF). For application: pneumococcal quality control serum	50	Strong overall correlation for most serotypes between MIA and ELISA. Correlation coefficient was 0.95 for serotypes 6B, 14, and 23F. It was ≧0.85 for 6 other serotypes (1, 4, 5, 9V, 18C, and 19F).
Balmer P, et al. [40] (2003)	UK	Cross-sectional	ELISA	9	Serum from at-risk group	47	Discrepancies in 21/47 (45%) were observed in a direct comparison between the two ELISA assays.
Lal G, et al. [24] (2005)	UK	Comparative	MIA vs. ELISA	9	U.S. reference standard serum (89SF); serum panel	120	Linear dynamic range over a 24-fold dilution. Did not show bead interference compared to monoplex and nonaplex assays. The assay specificity was <30% with heterologous inhibition and the sensitivity was high with the limit of detection ranging between 32.3 and 109.7 pg/mL. Inter- and intra-assay variability was low. Conjugated microspheres were stable over 12 months.
Marchese RD, et al. [33] (2006)	US	Comparative	In-house ELISA vs. standard ELISA	12	US reference standard serum (89SF)	120	The in-house ELISAs showed good agreement with the WHO ELISA.
Marchese RD, et al. [25] (2009)	US	Comparative	Multiplex ECL (pneumococcal electrochemiluminescence) assay vs. standard ELISA	23	US reference standard serum (89SF), WHO quality control panel, and Giebink sera	12	Met WHO criteria for all 7 serotypes. Overall correlation, r = 0.994.
Balloch A, et al. [41] (2010)	Australia, Finland, Fiji	Inter-lab comparison	ELISA	7	Well-characterized serum samples provided by Wyeth Vaccines, and sera from infants immunized PCV7 with and from healthy children over 2 years of age followed with a dose of PPSV23	24	Good agreement was found for the inter-laboratory comparison for most serotypes; differences in ELISA methodology influenced specific IgG measurement.
Whaley MJ, et al. [42] (2010)	UK, US	Comparative	3 MIAs	7	WHO-recommended standard reference and reference sera from vaccinated adults	11	MIAs generally showed higher antibody concentrations than ELISA but high variability for serotypes 6B, 18C, and 23F. None of the three assays met the current WHO criterion: 75% of sera within 40% of the assigned antibody concentrations for all seven serotypes (WHO).
Elberse KE, et al. [43] (2010)	Netherland	Comparative	MIA vs. ELISA	13	12 serum samples supplied by NIBSC; pre- and post PCV7 infant serum	188	Antibody concentrations by MIA were higher than those by ELISA. The protective threshold of 0.35 μg/mL should be reevaluated for use in the MIA. The threshold may need to be adjusted for each serotype.
Goldblatt D, et al. [44] (2011)	US, Finland	Comparative	Multiplex ECL vs. ELISA	7	U.S. reference standard serum (89SF), pediatric sera with and without vaccination of PCV7	50	Excellent concordance; 9 of 12 QC samples within ±40% of ELISA values.
Klein DL, et al. [35] (2012)	US	Comparative	MIA vs. ELISA	7	U.S. reference standard serum (89SF), paired maternal sera	50	<16% variability; cell wall polysaccharide (CWPs) content in pneumococcal serotype-specific capsular polysaccharides; assay is specific, robust, and reproducible and offers high throughput.
Zhang X, et al. [45] (2013)	US	Inter-lab comparison	MIA	14	Unpaired adult sera	57	High agreement in protective status classification across labs.
Balloch A, et al. [46] (2013)	Australia, Fiji	Comparative	MIA vs. ELISA	14	Infant and adult sera immunized with PCV7 and/or PPSV23	202	MIA poorly correlated with the ELISA, particularly for pre-immunization and infant samples.
Tan CY, et al. [47] (2018)	US, Germany, Japan	Comparative	MIA vs. ELISA	13	PCV7- or PCV13-immunized subject sera	1528	Robust linear correlation across all 13 serotypes. The protective level (0.35 μg/mL) is valid for 10 serotypes: 1, 3, 4, 6A, 7F, 9V, 14, 18C, 19F, and 23F.
Feyssaguet M, et al. [48] (2019)	Europe	Comparative	Multiplex ECL vs. ELISA	13	WHO standard serum (007sp); a panel of sera collected from infants and children vaccinated with PCV7, PHiD-CV, or PCV13 in 11 trials	452	This study assessed 2 different approaches for assessing the protective level of PCV7 serotypes: the ROC curve-based approach and Deming regression. The levels were approximated at 0.35 μg/mL (0.38 and 0.34 μg/mL, respectively). Individual thresholds for the PCV13 serotypes ranged between 0.24 and 0.51mg/mL across both approaches. The multiplex ECL assays were comparable to the WHO ELISA.
LaFon DC and Nahm MH [20] (2019)	US	Inter-lab comparison	MIA	23	Sera from healthy adults	10	Substantial inter-lab variability.
Meek B, et al. [39] (2019)	US, Sweden, Netherland, Denmark, UK, Iceland, Finland, Norway	Inter-lab comparison	MIA	13	WHO standard serum (007sp); 15 quality control samples distributed by Public Health England	13	MIA reproducible; some variability due to different conjugation methods, especially for serotype 4.
Nolan KM, et al. [38] (2020)	US	Bridging study	Multiplex ECL vs. ELISA	15	WHO standard serum (007sp)	128	All 15 serotypes met WHO criteria.

**Table 4:** Validation parameters and key findings of pneumococcal antibody assays.

Assay Type	Study	Serotype	Sample Type	WHO Validation Items *								Other Assessment Points
				Specificity	Accuracy	Precision	Detection Limit	Quantitation Limit	Linearity	Range	Robustness	
ELISA	Black S, et al. [30] (2000)	7	Infant sera (PCV7)	Not validation study								Efficacy assessed by surveillance of invasive pneumococcal disease; immunogenicity evaluated by IgG responses
	Marchese RD, et al. [33] (2006)	12	89SF	% Inhibition of homologous polysaccharides: >75%		Relative standard deviation: <30% for all serotypes	<2 ng/mL (each serotype)	<2 ng/mL (each serotype)				
	Lee H, et al. [34] (2017)	7	89SF and 5 calibration sera	Evaluation for 7 serotypes of PnPS antigens with optimal coating concentrations. The values of 5 calibration sera corresponded to each IgG value.	Good correlation between assigned values and observed values for all serotypes: r = 0.94–0.99	Reproducibility precision, CV (%): 1.65–8.24 (one exception: serotype 9V) Intermediate precision, CV(%): 1.88–20.32 except serotypes 4, 9V, and 14	Not found	0.05 μg/mL (serotype 4) to 0.093 μg/mL (serotype 19F)	Not found	1/250 to 1/24,414	Freeze–thaw stability, short-term temperature stability	Spiking recovery test
MIA	Pickering JW, et al. [28] (2002)	14	89SF	Homologous: ≧95% Heterologous: ≦15% for all 14 serotypes	Not found	Not found	Not found	Not found	Over seven 4-fold	1:20 to 1:81,920	Not found	9 serotypes were assessed by MIA and ELISA. Correlation coefficient: 0.95 (serotypes 6B, 14, 23F), >0.85 (serotypes 1, 4, 5, 9V, 18C, 19F)
	Biagini RE, et al. [23] (2003)	24	89SF	Proportion data not found. Homologous: 15 serotypes, Heterologous: 8 serotypes	Not found	Inter-assay CV (%): 13.2% from two independent microsphere preparations	Minimum detection concentration (MDC): 20 pg/mL (serotype 3) to 600 pg/mL (serotype 4)	Not found	Linearity across the dynamic range of this assay	1:100 to 1:316,000	Not found	Strong correlation with ELISA: r2 > 0.9994 ± 0.003 (*p* < 0.001)
	Lal G, et al. [24] (2005)	9	89SF	Homologous: > 90% Heterologous: <30% for all serotypes	Not found	Assay reproducibility, CV (%). Inter-assay variation from three separate assays: 12.1% (serogroup 19F) to 19.2% (serogroup 6B)	Limit of detection (LOD): 32.3 pg/mL (ST1) to 109.7 pg/mL (ST19F). LOD of ELISA: 5000 pg/mL	Limit of quantitation (LOQ): 64.6pg/mL (ST1) to 219.4pg/mL (ST19F)	Over 24-fold	1/20 to 1/81,920	Reagent stability, microsphere stability over 12 months	Good correlation with ELISA for all serotypes: r = 0.91 (serotypes 1 and 23F) and 0.96 (serotype 14)
	Klein DL, et al. [35] (2012)	7	89SF, WHO pneumococcal calibration human serum panel (n = 12)	Not found	6 out of 7 serotypes showed good correlation between MIA and the ELISA IgG level: serotype 14 (r2 > 0.99), serotype 19F (r2 = 0.94), serotype 23F (r2 = 0.91), and serotype 18C (r2 = 0.488).	Assay reproducibility, CV (%). Inter-assay variation from seven different dates: less than 16% for all serotypes. The mean intraplate CV: 6.2% (serotypes 18C and 23F) to 9.1% (serotype 5)	Not found	Not found	Not found	Not found (MIA) 1:20 to 1: 1280 (ELISA)	Day, operator, reagent lot differences	Good correlation with ELISA for all serotypes, r2 > 0.99 (serotype 14), r2 = 0.94 (19F), and r2 = 0.91 (23F), except 18C
	Whitelegg AME, et al. [36] (2012)	13	89SF, NHSBT sera (n = 193)	Serotype-specific IgG mean fluorescence intensities remained above 60% except for serotype 7F after preabsorption with each heterologous antigen.	Not found	Intra-assay coefficient of variation for specific IgG ranged between 4% and 25%. Inter-assay coefficient of variation was between 17% and 57%.	Not found	Not found	Not found	Not found	Not found	Assessment with NHSBT sera was implemented on pneumococcal IgG and IgM; IgG level of all 13 serotypes showed above WHO protective level
	Pavliakova D, et al. [37] (2018)	13	007sp	Homologous: ≧80% for all serotypes. Heterologous: <20% for all serotypes except serogroups 6 and 19.	The lower limits were 0.004 (serotype 3) to 0.032 (serotype 6A) ng/mL. The upper limits: 7250 (serotype 3) to 78,600 (serotype 6A) ng/mL.	Estimated assay variability due to day, analyst, and microsphere lot.	Not found	The lower limits of quantitation: 2 ng/mL (serotypes 1, 5, and 18C) to 38 ng/mL (serotype 19A) ng/mL	Linearity across the dynamic range	The most conservative assay dynamic range. The lower limits: 0.007 (serotype 3) to 0.071 (serotype 14) ng/mL. The upper limits: 7250 (serotype 3) to 68,207 (serotype 6A) ng/mL. Fold-difference > 500-fold	Day, primary and secondary antibody incubation times, temperature. Total variability: less than 20%, except serotype 6A	Bridging the WHO Pn PS ELISA
Multiplex ECL assay (MIA)	Marchese RD, et al. [25] (2009)	8	89SF, WHO sera (n = 12)	Homologous: ≧97% for all serotypes. Heterologous: <25% for all serotypes.	Not found	Precision (RSD): < 20% for each serotype on samples diluted between 1:1000 and 1:10,000, 22.6% to 41.7% on samples diluted at 1:100,000.	Lower limit of detection (adjusted at the 1:1000 minimum required dilution): 0.008 μg/mL (serotype 4) to 0.066 μg/mL (serotype 14)	Lower limit of quantification (adjusted at the 1:1000 minimum required dilution): 0.012 (serotype 3) to 0.1 (serotype 14) μg/mL	Not found	>100-fold	Day, analytes, reagent lot differences	Standard curve modeling, dilutability, effect of preadsorbent. 7 serotypes were assessed by ECL and ELISA. Correlation (r value): 0.885 (serotype 18C) to 0.997 (serotype 6B)
	Nolan KM, et al. [38] (2020)	15	007sp	Homologous: all samples met the acceptance criteria, ≧75% for all 15 serotypes. Heterologous: more than 87.5% of samples met the criteria, ≦25% for all serotypes.	% recovery range: 92 (serotype 3) to 122 (serotype 1) (acceptance criteria: 80 to 125%).	Intermediate precision (%GCV): 16.8 (serotype 18C) to 24.1 (serotype 7F) (acceptance criteria: <25%).	Limit of detection (LOD): 0.0007 μg/mL (serotype 3) to 0.0185 μg/mL (serotype 14) (acceptance criteria: ≦0.05).	Limit of quantitation (LOQ): 0.05 μg/mL (all serotypes except serotype 5) to 1.0 μg/mL (serotype 5) (acceptance criteria: low LOQ ≦ 0.1).	Dilutional linearity (fold bias per 10-fold dilution): 1.06 (serotype 19A) to 1.36 (serotype 22F) (acceptance criteria: less than ±2-fold per 10-fold increase in dilution)	1:1000 to 1:400,000	Not found	Selectivity, bridging the WHO Pn PS ELISA

* WHO validation items as given by the WHO Expert Committee on Biological Standardization (60th report) [18].

## Data Availability

Data related to this study are available upon a reasonable request.

## Supplementary Materials

The following supporting information can be downloaded at https://www.mdpi.com/article/10.3390/tropicalmed10080236/s1, File S1: Search Strategy. File S2: Included Studies. File S3: Preferred Reporting Items for Systematic reviews and Meta-Analyses extension for Scoping Reviews (PRISMA-ScR) Checklist. Refs. [56,57,58,59,60,61,67,68,69,70,71,72,73,74] are cited in Supplementary Materials.

## Abbreviations

CDC	Centers for Disease Control and Prevention
ELISA	Enzyme-linked immunosorbent assay
FDA	Food and Drug Administration
IgG	Immunoglobulin G
IPD	Invasive pneumococcal disease
LOD	Detection limit
LLOQ	Lower limit of quantification
LOQ	Quantitation limit
MIA	Multiplex immunoassay
PCR	Polymerase chain reaction
PCV	Pneumococcal conjugate vaccine
PCV7	7-valent pneumococcal conjugate vaccine
PCV10	10-valent pneumococcal conjugate vaccine
PCV13	13-valent pneumococcal conjugate vaccine
PPSV23	23-valent pneumococcal polysaccharide vaccine
PRISMA	Preferred Reporting Items for Systematic Reviews and Meta-analysis
RIA	Radioimmunoassay
ULOQ	Upper limit of quantification
WHO	World Health Organization
