# Supplementary material for: Quantitative Methods for Evaluating Antibody Responses to Pneumococcal Vaccines: A Scoping Review
Source: Trop Med Infect Dis. 2025 Aug 21;10(8):236. doi: 10.3390/tropicalmed10080236 (PMC12390409; doi:10.3390/tropicalmed10080236)
Supplement: Supplementary file 1 [file tropicalmed-10-00236-s001.zip › File S1.pdf]

File S1. Search Strategy

PubMed

Search ID	Query	Hits
1	Streptococcus pneumoniae [Title/Abstract]	20,798
2	pneumococc* [Title/Abstract]	20,848
3	Search 1 OR Search 2	32,110
4	Luminex [Title/Abstract]	5,605
5	multiplex* [Title/Abstract]	69,851
6	"ELISA" [Title/Abstract]	169,457
7	"enzyme linked immunosorbent assay" [Title/Abstract]	76,007
8	Search 4 OR Search 5 OR Search 6 OR Search 7	277,478
9	"pneumococcal IgG" [Title/Abstract]	73
10	"pneumococcal antibody" [Title/Abstract]	169
11	Search 9 OR Search 10	230
12	Search 3 AND Search 8	1,308
13	Search 11 OR Search 12	1,469

Scopus

Search ID	Query	Hits
1	Streptococcus pneumoniae (TITLE-ABS)	122,861
2	pneumococc* (TITLE-ABS)	94,902
3	Search 1 OR Search 2	175,923
4	Luminex (TITLE-ABS)	14,174
5	multiplex* (TITLE-ABS)	568,106
6	"ELISA" (TITLE-ABS)	323,229
7	"enzyme linked immunosorbent assay" (TITLE-ABS)	420,965
8	Search 4 OR Search 5 OR Search 6 OR Search 7	1,091,816
9	"pneumococcal IgG" (TITLE-ABS)	170
10	"pneumococcal antibody" (TITLE-ABS)	1,870
11	Search 9 OR Search 10	1,953
12	Search 3 AND Search 8	20,791
13	Search 11 OR Search 12	21,849
14	Search 13 with filter of Subject area and Keyword	2,946
