# Supplementary material for: Quantitative Methods for Evaluating Antibody Responses to Pneumococcal Vaccines: A Scoping Review
Source: Trop Med Infect Dis. 2025 Aug 21;10(8):236. doi: 10.3390/tropicalmed10080236 (PMC12390409; doi:10.3390/tropicalmed10080236)
Supplement: Supplementary file 1 [file tropicalmed-10-00236-s001.zip › File S2.pdf]

File S2. Included Studies

No.	Authors	Year	Title	Journal	DOI
1	Balloch A, Licciardi PV, Leach A, Nurkka A, Tang ML[41]	2010	Results from an inter-laboratory comparison of pneumococcal serotype-specific IgG measurement and critical parameters that affect assay performance	Vaccine	10.1016/j.vaccine.2009.11.011
2	Balloch A, Licciardi PV, Tang ML[46]	2013	Serotype-specific anti-pneumococcal IgG and immune competence: critical differences in interpretation criteria when different methods are used	J Clin Immunol	10.1007/s10875-012-9806-9
3	Balmer P, North J, Baxter D, Stanford E, Melegaro A, Kaczmarski EB, et al. [40]	2003	Measurement and interpretation of pneumococcal IgG levels for clinical management	Clin Exp Immunol	10.1046/j.1365-2249.2003.02232.x
4	Balmer P, Borrow R, Findlow J, Warrington R, Frankland S, Waight P, et al.[56] †	2007a	Age-stratified prevalences of pneumococcal-serotype-specific immunoglobulin G in England and their relationship to the serotype-specific incidence of invasive pneumococcal disease prior to the introduction of the pneumococcal 7-valent conjugate vaccine	Clin Vaccine Immunol	10.1128/CVI.00264-07
5	Balmer P, Cant AJ, Borrow R[67]	2007b	Anti-pneumococcal antibody titre measurement: what useful information does it yield?	J Clin Pathol	10.1136/jcp.2006.041210
6	Beck SC[19] †	2013	Making sense of serotype-specific pneumococcal antibody measurements	Ann Clin Biochem	10.1177/0004563213500241

7	Biagini RE, Schlottmann SA, Sammons DL, Smith JP, Snawder JC, Striley CA, et al. [23]	2003	Method for simultaneous measurement of antibodies to 23 pneumococcal capsular polysaccharides	Clin Diagn Lab Immunol	10.1128/cdli.10.5.744-750.2003
8	Black S, Shinefield H, Fireman B, Lewis E, Ray P, Hansen JR, et al. [30]	2000	Efficacy, safety and immunogenicity of heptavalent pneumococcal conjugate vaccine in children. Northern California Kaiser Permanente Vaccine Study Center Group	Pediatr Infect Dis J	10.1097/00006454-200003000-00003
9	Borrow R, Stanford E, Waight P, Helbert M, Balmer P, Warrington R, et al.[57] †	2008	Serotype-specific immune unresponsiveness to pneumococcal conjugate vaccine following invasive pneumococcal disease	Infect Immun	10.1128/IAI.00796-08
10	Concepcion NF, Frasch CE[50]*	2001	Pneumococcal type 22f polysaccharide absorption improves the specificity of a pneumococcal-polysaccharide enzyme-linked immunosorbent assay	Clin Diagn Lab Immunol	10.1128/cdli.8.2.266-272.2001
11	Daly TM, Pickering JW, Zhang X, Prince HE, Hill HR[53]*	2014	Multilaboratory assessment of threshold versus fold-change algorithms for minimizing analytical variability in multiplexed pneumococcal IgG measurements	Clin Vaccine Immunol	10.1128/cvi.00235-14
12	Daly TM, Hill HR[17] †	2015	Use and clinical interpretation of pneumococcal antibody measurements in the evaluation of humoral immune function	Clin Vaccine Immunol	10.1128/cvi.00735-14
13	Dunbar SA[29]	2023	Multiplexed suspension array immunoassays for detection of antibodies to pneumococcal polysaccharide and conjugate vaccines	Front Cell Infect Microbiol	10.3389/fcimb.2023.1296665

14	Elberse KE, Tcherniaeva I, Berbers GA, Schouls LM[43]	2010	Optimization and application of a multiplex bead-based assay to quantify serotype-specific IgG against Streptococcus pneumoniae polysaccharides: response to the booster vaccine after immunization with the pneumococcal 7-valent conjugate vaccine	Clin Vaccine Immunol	10.1128/cvi.00408-09
15	Feyssaguet M, Bellanger A, Nozay F, Friel D, Merck E, Verlant V, et al.[48]	2019	Comparison between a new multiplex electrochemiluminescence assay and the WHO reference enzyme-linked immunosorbent assay to measure serum antibodies against pneumococcal serotype-specific polysaccharides	Vaccine	10.1016/j.vaccine.2019.03.011
16	Goldblatt D, Ashton L, Zhang Y, Antonello J, Marchese RD[44]	2011	Comparison of a new multiplex binding assay versus the enzyme-linked immunosorbent assay for measurement of serotype-specific pneumococcal capsular polysaccharide IgG	Clin Vaccine Immunol	10.1128/cvi.05158-11
17	Gupta A, Mathad JS, Yang WT, Singh HK, Gupte N, Mave V, et al. [58] †	2014	Maternal pneumococcal capsular IgG antibodies and transplacental transfer are low in South Asian HIV-infected mother-infant pairs	Vaccine	10.1016/j.vaccine.2014.01.033
18	Hajjar J, Al-Kaabi A, Kutac C, Dunn J, Shearer WT, Orange JS[68] †	2018	Questioning the accuracy of currently available pneumococcal antibody testing	J Allergy Clin Immunol	10.1016/j.jaci.2018.06.013
19	Hansenová Maňásková S, van Belkum A, Endtz HP, Bikker FJ, Veerman EC, van Wamel WJ [52]*	2016	Comparison of non-magnetic and magnetic beads in bead-based assays	J Immunol Methods	10.1016/j.jim.2016.06.003

20	Haranaka M, Yono M, Kishino H, Igarashi R, Oshima N, Sawata M, et al.[57] †	2023	Safety, tolerability, and immunogenicity of a 21-valent pneumococcal conjugate vaccine, V116, in Japanese healthy adults: A Phase I study	Hum Vaccin Immunother	10.1080/21645515.2023.2228162
21	Hare ND, Smith BJ, Ballas ZK[59] †	2009	Antibody response to pneumococcal vaccination as a function of preimmunization titer	J Allergy Clin Immunol	10.1016/j.jaci.2008.09.021
22	Jiménez-Munguía I, van Wamel WJB, Rodríguez-Ortega MJ, Obando I[69]*	2017	Detection of Natural Antibodies and Serological Diagnosis of Pneumococcal Pneumonia Using a Bead-Based High-Throughput Assay	Methods Mol Biol	10.1007/978-1-4939-7180-0_13
23	Kanevsky I, Surendran N, McElwee K, Lei L, Watson W, Pride M, et al.[58] †	2023	Comparison of pneumococcal immunogenicity elicited by the PCV13 and PCV15 vaccines in adults 18 through 49 years of age	Vaccine	10.1016/j.vaccine.2023.09.043
24	Klein DL, Martinez JE, Hickey MH, Hassouna F, Zaman K, Steinhoff M[35]	2012	Development and characterization of a multiplex bead-based immunoassay to quantify pneumococcal capsular polysaccharide-specific antibodies	Clin Vaccine Immunol	10.1128/cvi.05535-11
25	LaFon DC, Nahm MH[59] †	2018a	Measuring immune responses to pneumococcal vaccines	J Immunol Methods	10.1016/j.jim.2018.08.002
26	LaFon DC, Nahm MH[26]	2018b	Measuring quantity and function of pneumococcal antibodies in immunoglobulin products	Transfusion	10.1111/trf.15015

27	LaFon DC, Nahm MH[20] †	2019	Interlaboratory variability in multiplexed pneumococcal antibody testing	J Allergy Clin Immunol	10.1016/j.jaci.2018.10.057
28	Lal G, Balmer P, Stanford E, Martin S, Warrington R, Borrow R[24]	2005	Development and validation of a nonaplex assay for the simultaneous quantitation of antibodies to nine Streptococcus pneumoniae serotypes	J Immunol Methods	10.1016/j.jim.2004.11.006
29	Lee H, Lim SY, Kim KH[34]	2017	Validation of the World Health Organization Enzyme-Linked Immunosorbent Assay for the Quantitation of Immunoglobulin G Serotype-Specific Anti-Pneumococcal Antibodies in Human Serum	J Korean Med Sci	10.3346/jkms.2017.32.10.1581
30	Linley E, Bell A, Gritzfeld JF, Borrow R[70] †	2019	Should Pneumococcal Serotype 3 Be Included in Serotype-Specific Immunoassays?	Vaccines (Basel)	10.3390/vaccines7010004
31	Marchese RD, Jain NT, Antonello J, Mallette L, Butterfield-Gerson KL, Raab J, et al.[33]*	2006	Enzyme-linked immunosorbent assay for measuring antibodies to pneumococcal polysaccharides for the PNEUMOVAX 23 vaccine: assay operating characteristics and correlation to the WHO international assay.	Clin Vaccine Immunol	10.1128/cvi.00014-06
32	Marchese RD, Jain NT, Antonello J, Mallette L, Butterfield-Gerson KL, Raab J, et al.[25]	2009	Optimization and validation of a multiplex, electrochemiluminescence-based detection assay for the quantitation of immunoglobulin G serotype-specific antipneumococcal antibodies in human serum	Clin Vaccine Immunol	10.1128/cvi.00415-08

33	Meek B, Ekström N, Kantsø B, Almond R, Findlow J, Gritzfeld JF, et al.[39]*	2019	Multilaboratory Comparison of Pneumococcal Multiplex Immunoassays Used in Immunosurveillance of Streptococcus pneumoniae across Europe	mSphere	10.1128/mSphere.00455-19
34	Nakashima K, Aoshima M, Ohfuji S, Yamawaki S, Nemoto M, Hasegawa S, et al.[60] †	2018	Immunogenicity of simultaneous versus sequential administration of a 23-valent pneumococcal polysaccharide vaccine and a quadrivalent influenza vaccine in older individuals: A randomized, open-label, non-inferiority trial	Hum Vaccin Immunother	10.1080/21645515.2018.1455476
35	Nolan KM, Zhang Y, Antonello JM, Howlett AH, Bonhomme CJ, Greway R, et al.[38]	2020	Enhanced antipneumococcal antibody electrochemiluminescence assay: validation and bridging to the WHO reference ELISA	Bioanalysis	10.4155/bio-2020-0023
36	Park MK, Briles DE, Nahm MH[49]*	2000	A latex bead-based flow cytometric immunoassay capable of simultaneous typing of multiple pneumococcal serotypes (multibead assay)	Clin Diagn Lab Immunol	10.1128/CDLI.7.3.486-489.2000
37	Park MA, Jenkins SM, Smith CY, Pyle RC, Sacco KA, Ryu E, et al.[71] †	2021	Pneumococcal serotype-specific cut-offs based on antibody responses to pneumococcal polysaccharide vaccination in healthy adults	Vaccine	10.1016/j.vaccine.2021.04.015
38	Pavliakova D, Giardina PC, Moghazeh S, Sebastian S, Koster M, Pavliak V, et al.[37]	2018	Development and Validation of 13-plex Luminex-Based Assay for Measuring Human Serum Antibodies to Streptococcus pneumoniae Capsular	mSphere	10.1128/mSphere.00128-18

39	Pickering JW, Larson MT, Martins TB, Copple SS, Hill HR[55] †	2010	Elimination of false-positive results in a luminex assay for pneumococcal antibodies	Clin Vaccine Immunol	10.1128/cvi.00329-09
40	Pickering JW, Martins TB, Greer RW, Schroder MC, Astill ME, Litwin CM, et al.[28] *	2002	A multiplexed fluorescent microsphere immunoassay for antibodies to pneumococcal capsular polysaccharides	Am J Clin Pathol	10.1309/lmch-c4q2-vfl9-3t1a
41	Platt HL, Cardona JF, Haranaka M, Schwartz HI, Narejos Perez S, Dowell A, et al.[56] †	2022	A phase 3 trial of safety, tolerability, and immunogenicity of V114, 15-valent pneumococcal conjugate vaccine, compared with 13-valent pneumococcal conjugate vaccine in adults 50 years of age and older (PNEU-AGE).	Vaccine	10.1016/j.vaccine.2021.08.049
42	Plikaytis BD, Goldblatt D, Frasch CE, Blondeau C, Bybel MJ, Giebink GS, et al.[27]	2000	An analytical model applied to a multicenter pneumococcal enzyme-linked immunosorbent assay study	J Clin Microbiol	10.1128/jcm.38.6.2043-2050.2000
43	Prins-van Ginkel AC, Berbers GA, Grundeken LH, Tcherniaeva I, Wittenberns JI, Elberse K, et al.[72] †	2016	Dynamics and Determinants of Pneumococcal Antibodies Specific against 13 Vaccine Serotypes in the Pre-Vaccination Era	PLoS One	10.1371/journal.pone.0147437
44	Schaballie H, Bosch B, Schrijvers R, Proesmans M, De Boeck K, Boon MN, et al.[54] †	2017	Fifth Percentile Cutoff Values for Antipneumococcal Polysaccharide and Anti-Salmonella typhi Vi IgG Describe a Normal Polysaccharide Response	Front Immunol	10.3389/fimmu.2017.00546
45	Schlottmann SA, Jain N, Chirmule N, Esser MT[51] *	2006	A novel chemistry for conjugating pneumococcal polysaccharides to Luminex microspheres	J Immunol Methods	10.1016/j.jim.2005.11.019

46	Siber GR, Chang I, Baker S, Fernsten P, O'Brien KL, Santosham M, et al. [32] †	2007	Estimating the protective concentration of anti-pneumococcal capsular polysaccharide antibodies	Vaccine	10.1016/j.vaccine.2007.01.119
47	Sorensen RU, Leiva LE[60]*	2014	Measurement of pneumococcal polysaccharide antibodies	J Clin Immunol	10.1007/s10875-013-9977-z
48	Sorensen RU, Edgar JDM[73] †	2018	Overview of antibody-mediated immunity to S. pneumoniae: pneumococcal infections, pneumococcal immunity assessment, and recommendations for IG product evaluation	Transfusion	10.1111/trf.15044
49	Tan CY, Immermann FW, Sebastian S, Pride MW, Pavliakova D, Belanger KA, et al.[47] †	2018	Evaluation of a Validated Luminex-Based Multiplex Immunoassay for Measuring Immunoglobulin G Antibodies in Serum to Pneumococcal Capsular Polysaccharides	mSphere	10.1128/mSphere.00127-18
50	Wernette CM, Frasch CE, Madore D, Carlone G, Goldblatt D, Plikaytis B, et al. [16]	2003	Enzyme-linked immunosorbent assay for quantitation of human antibodies to pneumococcal polysaccharides	Clin Diagn Lab Immunol	10.1128/cdli.10.4.514-519.2003
51	van Westen E, Knol MJ, Wijmenga-Monsuur AJ, Tcherniaeva I, Schouls LM, Sanders EAM, et al.[61] †	2018	Serotype-specific igg antibody waning after pneumococcal conjugate primary series vaccinations with either the 10-valent or the 13-valent vaccine	Vaccines	10.3390/vaccines6040082
52	Whaley MJ, Rose C, Martinez J, Laher G, Sammons DL, Smith JP, et al.[42] †	2010	Interlaboratory comparison of three multiplexed bead-based immunoassays for measuring serum antibodies to pneumococcal polysaccharides	Clin Vaccine Immunol	10.1128/cvi.00022-10

53	Whitelegg AME, Birtwistle J, Richter A, Campbell JP, Turner JE, Ahmed TM, et al.[36]	2012	Measurement of antibodies to pneumococcal, meningococcal and haemophilus polysaccharides, and tetanus and diphtheria toxoids using a 19-plexed assay	J Immunol Methods	10.1016/j.jim.2012.01.007
54	Zhang X, Simmerman K, Yen-Lieberman B, Daly TM[45] †	2013	Impact of analytical variability on clinical interpretation of multiplex pneumococcal serology assays	Clin Vaccine Immunol	10.1128/cvi.00223-13
55	Zimmermann P, Ritz N, Perrett KP, Messina NL, van der Klis FRM, Curtis N[74] †	2021	Correlation of Vaccine Responses	Front Immunol	10.3389/fimmu.2021.646677

* Studies included in Group 4 - Description on Development (Adsorption, Conjugation, Evaluation Methods)
† Studies included in Group 5 – Interpretation and application of findings
